# Gender‐Based Cyberviolence Prevention in European Secondary Education: A Scoping Review on the Intersection Between Comprehensive Sexuality Education and Digital Literacy

**DOI:** 10.1111/josh.70221

**Published:** 2026-08-25

**Authors:** Valeria Piras, Arpita Chakraborty

**Affiliations:** ^1^ Institute for Educational Technology, National Research Council of Italy Genoa Italy; ^2^ Institute for Research on Genders and Sexualities, Dublin City University Dublin Ireland

**Keywords:** comprehensive sexuality education, digital literacy education, European Union, gender‐based cyberviolence, scoping review, secondary education

## Abstract

**Background:**

The increasing pervasiveness of digital technologies in adolescent life has heightened concerns about gender‐based cyberviolence, especially toward girls, LGBTQI+ youth, and other marginalized groups. While European Union (EU) policy, including Directive (EU) 2024/1385, recognizes these harms, formal education systems in EU Member States lack integrated strategies that connect Comprehensive Sexuality Education (CSE) and Digital Literacy (DL).

**Methods:**

This scoping review explores how academic research addresses this intersection in secondary education, drawing on 14 peer‐reviewed studies published between 2014 and 2025 and sourced from Scopus, Web of Science, and Research Rabbit.

**Findings:**

The review reveals a fragmented field: CSE and DL are often treated in isolation, and cyberviolence prevention is rarely included. Young people's perspectives remain largely absent.

**Implications for School Health Policy, Practice, and Equity:**

These gaps highlight a misalignment between policy ambitions and educational practices. This review offers insight into how health education initiatives in schools can be reoriented to better integrate comprehensive sexuality education and digital literacy, thereby contributing to the prevention of gender‐based online harm.

**Conclusions:**

Integrating CSE and DL in secondary schools is essential to support more equitable, policy‐aligned, and effective prevention of gender‐based cyberviolence among adolescents.

## 1 Introduction

The rapid growth of digital technologies has significantly shaped adolescents' social interactions, bringing both opportunities and risks. Gender‐based cyberviolence has become a major concern, especially for girls, LGBTQI+ youth, and other marginalized groups facing intensified forms of offline oppression in digital spaces. Although European (referring specifically to countries that are member states of the European Union at the time of analysis) institutions have acknowledged the need for preventive action, formal education systems remain unprepared. Secondary school curricula often treat sexuality education and digital literacy as separate, overlooking their intersection. Comprehensive Sexuality Education (CSE), grounded in human rights and gender equality, promotes autonomy, consent, and emotional awareness [1, 2, 3, 4]. Yet these factors are increasingly negotiated within digitally mediated environments, making the integration with digital literacy essential.

The intersection between Comprehensive Sexuality Education (CSE) and digital literacy is increasingly essential in the European secondary education context, particularly in light of emerging policy frameworks such as Directive (EU) 2024/1385 [5] on combating violence against women and domestic violence. Articles 5 to 8 of the Directive explicitly recognize forms of gender‐based cyberviolence, including cyberstalking, online harassment, and the non‐consensual sharing of intimate material, as serious and distinct manifestations of violence that require preventive and educational responses. These behaviors occur precisely at the convergence of sexuality, gendered power relations, and digitally mediated interaction. Adolescents today experience intimacy, peer relationships, sexual socialization, and identity formation within online environments shaped by platform cultures, algorithmic exposure, and digitally circulating sexual content. Consequently, sexuality education that remains limited to offline relationships risks failing to address the realities of digital consent, coercion, and gendered harm. At the same time, digital literacy initiatives often adopt a technical or safety‐oriented approach, focusing on privacy or cyberbullying while neglecting the relational, sexual, and gendered dimensions of online violence. Integrating CSE and digital literacy is therefore not merely complementary but necessary to equip young people with critical, rights‐based competencies to navigate digital intimacy, recognize abusive dynamics, and prevent gender‐based cyberviolence in line with EU legal and educational commitments.

However, CSE and digital literacy are rarely integrated in EU schools, neglecting key issues like online consent and digital gendered power. This scoping review examines how academic literature addresses this intersection in EU secondary education, identifying gaps and calling for more cohesive, integrative approaches.

## 2 Background

Rapid technological developments in computers, smartphones and tracking devices have provided perpetrators of domestic and family violence with new tools to harm, threaten and abuse their victims. This includes acts such as posting personal images online, online impersonation that damages or undermines the victim's reputation, hacking or accessing the victim's mail or social media and banking accounts or limiting the victim's access to technology or the internet [6, 7, 8]. These forms of abuse are commonly referred to as technology‐facilitated abuse and include technology‐facilitated sexual violence, encompassing behaviors in which digital tools enable both virtual and offline sexually based harms [9, 10].

A range of concepts has been developed to describe the intersection between gender‐based violence and technology. However, despite the emergence of these definitions and the growing awareness of gender‐based cyberviolence, formal education systems across Europe continue to lack cohesive, sustainable, and inclusive strategies to address it. Gender‐based cyberviolence is an increasingly pervasive phenomenon, particularly affecting adolescents, especially girls, LGBTQI+ youth, and other marginalized groups, who engage in complex relational practices within digital spaces that often reproduce and amplify offline inequalities [11, 12, 13]. Its consequences are psychological, social, and political, undermining young people's access to safety, dignity, and democratic participation [14, 15, 16].

### 2.1 Gender‐Based Cyberviolence in Education

European institutions, including the Fundamental Rights Agency (FRA) and the European Institute for Gender Equality [17], have repeatedly emphasized the urgent need to address gender‐based cyberviolence through preventive education.

According to FRA [18], 20% of women aged 18–29 have experienced cyber‐sexual harassment, understood as unwanted sexualized behaviors enacted through digital technologies, such as unsolicited sexual messages, non‐consensual image sharing, or online intimidation, which often occurs alongside physical and sexual abuse. More recent evidence confirms the persistence and pervasiveness of these forms of violence in digital spaces. For instance, Plan International's report [19] found that 58% of girls and young women aged 15–25 across 31 countries had faced online harassment or abuse, with half of respondents reporting that they experience more harassment online than in public spaces. At the European level, data collected [20] based on interviews with over 42,000 women aged 18–74 across all EU Member States plus the United Kingdom represents one of the most recent attempts to capture cyber violence against women in a comparative way. The survey found that 11% of women and girls had experienced cyber harassment since the age of 15, while 5% had experienced cyber stalking since the same age.

Despite this, it remains difficult to find systemic educational programs that fully address online and technology‐facilitated gender‐based violence. Where prevention programs exist, they typically prioritize individual safety or digital etiquette, overlooking deeper issues such as gender norms, emotional dynamics, and power relations within digital environments [21, 22].

Comprehensive Sexuality Education (CSE) covers topics such as sexual and reproductive health, relationships, consent, gender equality, and sexual orientation, and recognizes how power dynamics and gender norms shape sexuality [2]. Although international guidelines recommend starting CSE in early childhood and continuing through adolescence, its implementation remains uneven across Europe [4, 23]. In many cases, it is non‐mandatory, contested, or delegated to local actors, leading to inconsistent coverage [24]. Some countries offer comprehensive, relational models, while others adopt risk‐based or abstinence‐focused approaches [4, 25, 26], often neglecting online behaviors, digital consent, or cyber‐intimacy. Though cyberviolence is mentioned in UNESCO's guidance [2], its specific manifestations, such as online sexual harassment, image‐based abuse, or technology‐facilitated coercive control, are not explored in depth, nor is it accompanied by detailed pedagogical frameworks or practical tools for educators, and current practices still fall short of addressing digital gender‐based risks among adolescents.

Digital education shows similar concerns. Children increasingly rely on AI chatbots for academic and emotional support [27], while exposure to unregulated online media shapes their sexual behaviors [28]. These trends raise growing concerns about the impact of digital content on youth.

Despite the Digital Education Action Plan 2021–2027 emphasizing the importance of digital literacy and responsible technology use [29], gendered dimensions of digital life, such as online misogyny, digital consent, and gender‐based cyberviolence, remain largely absent from school curricula [21]. Although Directive (EU) 2024/1385 explicitly recognizes cyberstalking, online harassment, and the non‐consensual sharing of intimate material, educational systems have yet to translate these priorities into concrete curricular content, teacher training, or prevention strategies. Structured educational approaches to address these issues are still rare, and civil society organizations working at the intersection of gender, technology, and education often encounter institutional resistance [17, 22]. The lack of disaggregated, comparable data across EU Member States further limits evidence‐based policymaking. This disconnect between policy imperatives and school‐level implementation is especially troubling given the growing influence of online spaces that promote misogyny and toxic masculinity, particularly among adolescent boys and young men [30, 31].

### 2.2 The Need for a Scoping Literature Review

Given the urgency of gender‐based cyberviolence, especially in adolescent digital life, there is a pressing need to understand how academic research addresses its educational dimensions in EU secondary schools (ages 11–19). Despite digital spaces becoming central to gendered harm, the role of education in responding remains underexplored in scholarly literature. This scoping review maps current academic engagement at the intersection of Comprehensive Sexuality Education (CSE) and Digital Literacy Education, identifying conceptual, thematic, and geographic gaps. It assesses whether research supports the development of integrative, rights‐based, and digitally aware pedagogical frameworks. In the fragmented European educational landscape, marked by uneven implementation of sexuality education and varied digital literacy approaches, this review aims to inform both scholarship and inclusive educational strategies aligned with EU values and policy priorities.

## 3 Method

### 3.1 Protocol and Registration

This scoping review followed the PRISMA‐ScR [32] framework to ensure methodological rigor and transparency. An internal protocol guided the process, though it was not formally registered or publicly shared.

### 3.2 Eligibility Criteria

This review included peer‐reviewed studies published between January 2014 and June 2025 that addressed comprehensive sexuality education and digital/media literacy. The focus was on European Union Member States, aligning with EU values on inclusion, well‐being, and youth rights [33], and with UNESCO's guidance [2] on sexuality education as a contributor to learners' health and dignity. Given that this review is situated within the European Union policy context, particularly Directive (EU) 2024/1385, geographical eligibility was defined to capture literature relevant to educational practice within EU secondary schools. Studies were included when they (a) were conducted in EU Member States, (b) analyzed EU‐based school curricula or educational interventions, or (c) explicitly addressed European institutional or curricular contexts, even when empirical data were not limited to a single country. References to EU Member States and European educational frameworks were used as additional indicators of contextual relevance during screening. No language restrictions were applied in the initial search.

### 3.3 Information Sources

The literature search was conducted in Scopus and Web of Science and complemented by citation mapping using Research Rabbit, with the final search completed on July 16, 2025.

### 3.4 Search Strategy

Scopus and Web of Science were searched using a two‐step strategy designed to capture both policy‐relevant and broader academic literature. The first step targeted studies explicitly addressing the intersection of comprehensive sexuality education, digital literacy, and gender‐based cyberviolence, using terminology aligned with Articles 5–8 of Directive (EU) 2024/1385. The second step broadened the scope to include literature examining the integration of comprehensive sexuality education and digital literacy in school settings. Together, these steps enabled a systematic mapping of both focused and general educational approaches to digitally mediated gender‐based harm.

Initial broad queries combining comprehensive sexuality education, digital/media literacy, and cyberviolence yielded no usable results in Scopus due to syntax errors, such as the platform rejecting terms like “manosphere” and suggesting unrelated replacements like “nanosphere”. In a second phase, the focus was broadened to explore more general intersections between CSE and digital literacy in secondary education.

The database search yielded 14 records in Scopus and 12 in Web of Science, which were de‐duplicated and screened for relevance, resulting in 10 studies retained from Scopus and Web of Science for full‐text assessment and preliminary charting.

Additional sources were identified through citation‐based mapping using Research Rabbit, applying the same eligibility criteria. The AI feature of Research Rabbit automatically prompted 86 suggestions of similar scholarly works, 24 earlier works, and 12 later works. The full search strategy, including database‐specific search strings, applied limits, and records retrieved, is reported in Table 1.

**TABLE 1:** Detailed search strategy conducted on Scopus, Web of Science and Research Rabbit.

Database/source	Search string/query	Filters/limits	Records retrieved
Scopus	**Step 1:** TITLE‐ABS‐KEY((“comprehensive sexuality education” OR “sexuality education” OR “sex education”) AND (“digital literacy” OR “digital competence” OR “online literacy” OR “digital skills” OR “digital education” OR “media literacy”) AND (“gender‐based violence” OR “cyber violence” OR “online violence” OR “online harassment” OR “cyber harassment” OR “online misogyny” OR “cyber‐bullying”)) AND PUB YEAR > 2014 AND PUB YEAR < 2025	2014–2025; peer‐reviewed; European institutions.	0
Web of Science	**Step 1:** TS = ((“comprehensive sexuality education” OR “sexuality education” OR “sex education”) AND (“digital literacy” OR “digital competence” OR “online literacy” OR “digital skills” OR “digital education” OR “media literacy”) AND (“gender‐based violence” OR “cyber violence” OR “online violence” OR “online harassment” OR “cyber harassment” OR “online misogyny” OR “cyber‐bullying”)) AND PUB YEAR > 2014 AND PUB YEAR < 2025	2014–2025; peer‐reviewed; European institutions.	0
Scopus	**Step 2:** TITLE‐ABS‐KEY((“comprehensive sexuality education” OR “sexuality education” OR “sex education”) AND (“digital literacy” OR “media literacy” OR “digital competence” OR “online literacy” OR “digital education” OR “digital skills”)) AND PUB YEAR > 2014 AND PUB YEAR < 2025	2014–2025; peer‐reviewed; European institutions.	14
Web of Science	**Step 2:** TS = ((“comprehensive sexuality education” OR “sexuality education” OR “sex education”) AND (“digital literacy” OR “media literacy” OR “digital competence” OR “online literacy” OR “digital education” OR “digital skills”)) AND PUB Y EAR > 2014 AND PUB YEAR < 2025	2014–2025; peer‐reviewed; European institutions.	12
Research Rabbit	No search string was used. The Scopus and Web of Science record collection was imported into Research Rabbit and used as the seed set for citation‐based mapping and similarity‐based recommendations.	2014–2025; peer‐reviewed; European institutions.	86

### 3.5 Selection of Sources of Evidence

After de‐duplication and EU‐focused screening of Scopus and Web of Science records, titles and abstracts were reviewed, excluding studies outside secondary education (ages 11–19) or the scope of sexuality education and digital/media literacy, yielding 10 articles for full‐text review. Citation‐based mapping using Research Rabbit focused on later publications, identifying 12 relevant works from a shared collection of 86 database articles and generating 35 additional suggestions, from which 7 articles were selected based on publication date, thematic relevance, and EU focus. Overall, 14 peer‐reviewed articles were included.

### 3.6 Data Items Analysis and Charting Process

Two independent reviewers conducted the data analysis: one extracted data from Scopus and Web of Science, the other from Research Rabbit. After an initial screening using predefined eligibility criteria, selected references were compiled into a single Excel file.

The charting form included the following fields: Type, Authors, Title, Publication Title, Abstract, Place, and Year. Four analytical columns were also added to assess whether each article:

1. Addresses Comprehensive Sexuality Education (CSE).
2. Engages with digital education or media literacy.
3. Explicitly mentions gender‐based cyberviolence or technology‐facilitated abuse.
4. Describes or analyses educational interventions in secondary school settings (e.g., case studies, pilot programs).

Each publication was evaluated against the four analytical dimensions using its abstract and full text, where available. Only studies addressing at least two of the four criteria were included in the final synthesis. This ensured a meaningful focus on how CSE, digital literacy, and gender‐based cyberviolence prevention intersect within secondary education in EU Member States. In the final stage, both reviewers jointly reviewed all entries in the Excel sheet to minimize bias and ensure consistency through double‐check validation.

### 3.7 Critical Appraisal of Individual Sources of Evidence

As is typical for scoping reviews, no formal critical appraisal or risk of bias assessment was performed. Nevertheless, by including only peer‐reviewed academic literature, a baseline level of methodological rigor was ensured.

### 3.8 Synthesis of Results

Among the 10 studies retained for analytical charting from Scopus and Web of Science:

- 5 articles explicitly addressed comprehensive sexuality education, or sexuality education.
- 6 articles discussed aspects of digital or media literacy.
- 4 articles described or evaluated concrete educational interventions.
- And only 1 article explicitly addressed cyberviolence or related digital risks.

Although each analytical category was similarly represented, the specific articles fulfilling them varied widely, highlighting the fragmented nature of the literature. Few studies addressed multiple dimensions in an integrated way. Cross‐referencing datasets helped identify overlaps, revealing that only one article [34] addressed three out of four core themes. While it did not explicitly refer to CSE, it focused on digital/media literacy and reported empirical findings on non‐consensual sexting and consent‐related educational gaps, recommending the inclusion of these topics in media literacy and anti‐bullying programs.

To ensure analytical coherence, the final synthesis concentrated on the 7 studies addressing at least two of the four dimensions, enabling a more focused examination of contributions engaging with the intersection of sexuality education and digital/media literacy in EU secondary schools, despite the limited evidence base.

**FIGURE 1:**
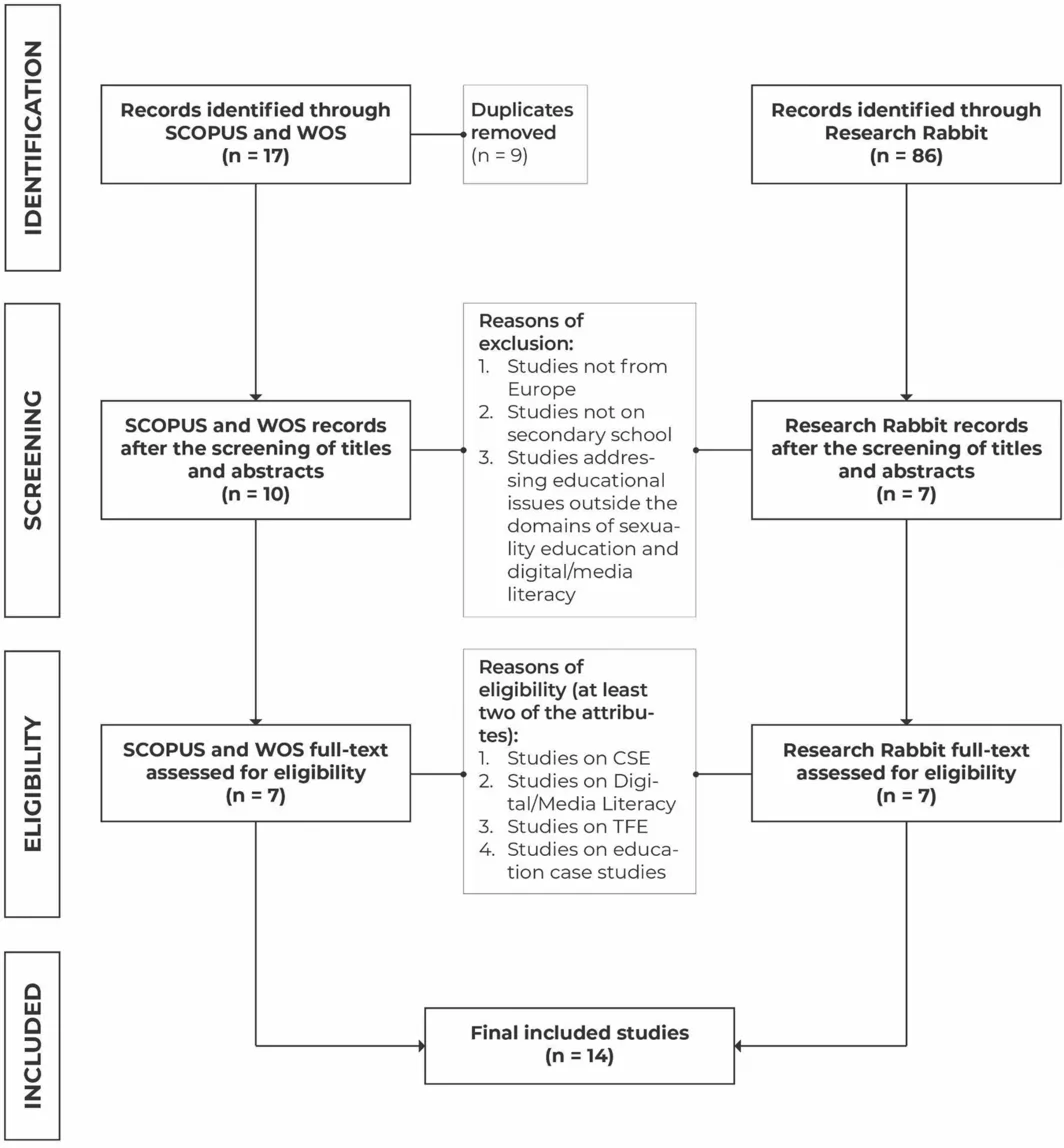
Flowchart of the scoping review process.

The exploratory phase using Research Rabbit produced additional sources that complemented and, in some cases, broadened the thematic scope of the initial dataset. These results were analyzed using the same four analytical dimensions as those applied to Scopus and Web of Science to ensure consistency and comparability (see Figure 1). Among the 7 studies selected from Research Rabbit:

- 6 articles explicitly addressed comprehensive sexuality education, or sexuality education.
- 4 articles discussed aspects of digital or media literacy.
- 3 articles described or evaluated concrete educational interventions.
- And only 1 article explicitly addressed cyberviolence or related digital risks.

## 4 Results

Results are presented in two stages to reflect the sequential identification process: first, the primary database search conducted in Scopus and Web of Science; second, the complementary citation‐based mapping conducted in Research Rabbit. However, records from both stages were screened using the same eligibility criteria and analyzed according to the same four predefined dimensions. The final synthesis included 14 studies in total: 7 identified through Scopus/Web of Science and 7 identified through Research Rabbit.

After the source‐specific results are presented, Table 2 provides a consolidated overview of the characteristics and thematic categorization of all 14 studies included in the final synthesis.

**TABLE 2:** Characteristics and thematic categorization of the 14 studies included in the final synthesis, by source of identification.

							The article addresses the topic of			
Source	Type	Authors	Title	Publication title (if in a Journal or a Book)	Place	Year	Comprehensive sexuality education	Digital education or media literacy	Gender‐based cyberviolence or technology‐facilitated abuse	Educational interventions in secondary school settings (e.g., case studies, pilot programs)
Scopus/Web of Science	Book chapter	Rojas‐Estrada, E.G.; et al.	Sexual (Mis)information: Pornography and Adolescence in the Digital Space	Comprehensive Sexuality Education for Gender‐Based Violence Prevention	USA, Spain	2024	YES	YES	NO	NO
Scopus/Web of Science	Journal Article	Barriuso‐Ortega, S.; et al.	Analysis of Sex‐Education Programs for Teenagers in Spain and Other Countries	Revista Electronica Educare	Spain	2022	YES	NO	NO	YES
Scopus/Web of Science	Journal Article	Prokop‐Dorner, A.; et al.	Teaching methods for critical thinking in health education of children up to high school: A scoping review	PLOS ONE	Poland	2024	YES	NO	NO	YES
Scopus/Web of Science	Journal Article	Delisle, B.	The change of youth sexuality. First Love 2005–2022—How can we reach the youth today; [Jugendsexualität im Wandel ?]	Padiatrische Praxis	Germany	2023	YES	YES	NO	NO
Scopus/Web of Science	Journal Article	Calderón‐Sandoval, O.; et al.	Debating Sexual Consent in the Teen Series The Hockey Girls: Reactions of Instagram Audiences	Sex Education	Spain	2024	YES	YES	NO	NO
Scopus/Web of Science	Journal Article	Testa, G.; et al.	Problematic Pornography Use in Adolescents: From Prevention to Intervention	Current Addiction Reports	Spain	2023	NO	YES	NO	YES
Scopus/Web of Science	Journal Article	Boer, S.; et al.	Prevalence and Correlates of Sext‐Sharing Among a Representative Sample of Youth in the Netherlands	Frontiers in Psychology	Netherlands	2021	NO	YES	YES	YES
Research Rabbit	Journal Article	Vandenbosch, L.; et al.	The Relationship Between Online Pornography and the Sexual Objectification of Women: The Attenuating Role of Porn Literacy Education	Journal of Communication	Netherlands	2017	NO	YES	NO	YES
Research Rabbit	Journal Article	Cegolon, L.	Birth control knowledge among freshmen of four Italian universities	Scientific Reports	Italy	2020	YES	YES	NO	NO
Research Rabbit	Journal Article	Sill, J. M.	“I wouldn't have ever known, if it wasn't for porn” – LGBT+ university students' experiences of sex and relationships education, a retrospective exploration	Sexuality, Society and Learning	UK	2022	YES	YES	NO	NO
Research Rabbit	Book chapter	Banks, J.; et al.	The role of technology in stalking and coercive control among young people	Young People, Stalking Awareness and Domestic Abuse	UK	2023	YES	NO	YES	NO
Research Rabbit	Journal Article	Dawson, K.; et al.	Development of a Measure to Assess What Young Heterosexual Adults Say They Learn About Sex from Pornography	Archives of Sexual Behavior	Ireland	2022	YES	YES	NO	NO
Research Rabbit	Journal Article	Roien, L. A.; et al.	From Deviance to Diversity: Discourses and Problem atisations in Fifty Years of Sexuality Education in Denmark	Sex Education	Denmark	2022	YES	NO	NO	YES
Research Rabbit	Journal Article	Seiler‐Ramadas, R.; et al.	Adolescents' Perspective on Their Sexual Knowledge and the Role of School in Addressing Emotions in Sex Education: An Exploratory Analysis of Two School Types in Austria	The Journal of Sex Research	Austria	2020	YES	NO	NO	YES

### 4.1 Results on Scopus and Web of Science

Following screening and selection, 17 unique records were identified across Scopus and Web of Science. After applying EU‐focused geographic filters and screening abstracts and full texts for relevance to secondary school‐aged youth (approximately 11–19 years), 10 articles were retained from the Scopus and Web of Science search for full‐text assessment and preliminary charting. These studies varied in design and thematic focus and were analyzed across four key dimensions: comprehensive sexuality education, digital or media literacy, gender‐based cyber violence or technology‐facilitated abuse, and educational interventions in secondary school settings.

Of these 10 articles, 7 met the final analytical inclusion threshold, defined as addressing at least two of the four predefined dimensions, and were therefore included in the final synthesis. Within the Scopus and Web of Science subset, five articles explicitly addressed comprehensive sexuality education, five engaged with digital or media literacy, and four described or evaluated educational interventions. Only one study explicitly referred to cyber violence or related phenomena such as sexting or non‐consensual image sharing.

Although the studies were relatively evenly distributed across themes, most addressed only two dimensions, with only one covering three and none encompassing all four, revealing a lack of studies addressing sexuality education, digital/media literacy, and violence prevention in an integrated way.

Complementary sources identified through Research Rabbit were analyzed using the same framework to expand the literature mapping.

### 4.2 Results on Research Rabbit

Research Rabbit complemented the Scopus and Web of Science search by identifying 86 unique later works related to the initial dataset. After de‐duplication and EU‐focused geographic screening, 7 sources remained. Following abstract screening and full‐text review, all 7 studies, focused on secondary school‐aged youth (approximately 11–19 years), wereincluded in the final sample. The selected studies varied in design and thematic focus and were analyzed using the same four key dimensions as in the first stage: six explicitly addressed comprehensive sexuality education, four engaged with digital or media literacy, and three described or evaluated educational interventions. Only one book chapter explicitly referred to cyberviolence or related phenomena such as stalking and coercive control. Overall, these findings reinforce the results of the first review stage, confirming the lack of integrated approaches linking sexuality education, digital/media literacy, and violence prevention in European secondary education research. Notably, one longitudinal study involving 1947 participants aged 13–25 demonstrated that increased porn literacy education weakened the association between exposure to sexually explicit internet material and sexist attitudes, underscoring the importance of integrating media literacy and sexuality education.

## 5 Discussion

The findings of this scoping review reveal a notable absence of cohesive and integrated research addressing the intersection of comprehensive sexuality education (CSE) and digital literacy within European secondary education. Although individual components, such as CSE, digital/media literacy, and educational interventions, are present across several studies, their systematic integration remains rare, and explicit attention to gender‐based cyberviolence is even more marginal. While a limited number of studies propose integrating content related to consent and online behavior within media literacy frameworks, such proposals tend to remain narrow in scope and insufficiently aligned with the complexity of digitally mediated gender‐based harm.

This gap is particularly salient in light of Articles 5–8 of Directive (EU) 2024/1385, which conceptualize gender‐based cyberviolence as a complex phenomenon requiring comprehensive educational responses. However, current research does not reflect this complexity nor provide integrated educational strategies aligned with EU policy ambitions.

This fragmentation may be partly explained by the interdisciplinary nature of the topic and by uneven CSE implementation across EU Member States, where provision is often non‐mandatory and locally delegated.

Beyond this general fragmentation, the reviewed literature reveals clear thematic patterns in how sexuality education and digital media are currently conceptualized. Across several studies, a consistent finding concerns the role of online pornography as a central, yet largely unmediated, source of sexual learning for adolescents and young adults. Approximately one third of the reviewed contributions (5 out of 14) explicitly address pornography in relation to sexuality education and digital media use, highlighting its growing relevance within young people's sexual socialization. In contexts where comprehensive sexuality education is limited, non‐mandatory, or insufficiently inclusive, pornography often emerges as a form of self‐directed sexual education, filling an educational vacuum left by formal institutions [35, 36].

Several studies critically examine the consequences of unrestricted or poorly regulated access to pornographic content among young people. In particular, the absence of structured educational guidance and regulatory frameworks may contribute to the internalization of stereotypical gender roles, unrealistic body standards, and distorted understandings of sexuality, intimacy, and consent [35, 37, 38]. These works underscore how exposure to pornographic representations, when not accompanied by critical media and sexuality education, can reinforce unequal power relations and normative sexual scripts that conflict with rights‐based approaches to sexuality education.

Other contributions further highlight that, in the absence of comprehensive and inclusive sexuality education, pornography may become one of the few accessible sources of information about sex and relationships. This dynamic is particularly evident among LGBTQ+ young people [36], whose experiences of formal sex education are often described as heteronormative, exclusionary, or irrelevant, leading them to rely on online content and pornography as alternative learning resources. Complementing these qualitative insights, empirical evidence shows that young adults report learning from pornography information related to sexual performance, body esthetics, and sexual exploration [39]. However, such learning is uneven, frequently incomplete, and rarely embedded within frameworks that emphasize mutual consent, emotional wellbeing, and gender equality.

These findings must be situated within a broader digital information ecosystem in which the internet and social media have become primary sources of sexual knowledge, particularly in national contexts where school‐based sexuality education is fragmented or non‐mandatory [40]. While media literacy is frequently identified as a necessary response to misinformation and harmful representations [35], the reviewed literature suggests that technical, health‐ or risk‐focused approaches alone are insufficient. While pornography and sexualized online content are digitally mediated phenomena, their influence extends beyond media consumption into the production of sexual knowledge, relational norms, and power dynamics; addressing these issues solely through content restriction or isolated media literacy interventions therefore risks underestimating their formative role in adolescents' understandings of consent, intimacy, and gender relations.

In addition, the review highlights the severely limited engagement of children and young people themselves in the development of sexuality education policies related to digital content. Although the literature frequently documents how adolescents use digital media in their everyday lives, young people are rarely consulted regarding how sexuality education and digital learning could better reflect their needs, experiences, and perceptions of safety.

Taken as a whole, these findings suggest that the limited presence of research explicitly addressing the intersection of comprehensive sexuality education, digital literacy, and gender‐based cyberviolence constitutes a finding in itself. This was already evident during the search process, where the underrepresentation of key terms, such as those related to online misogyny and the manosphere, highlighted the marginal position these issues continue to occupy within mainstream educational research. The scarcity of integrated studies, despite the clear relevance of these phenomena in adolescents' digital lives and their prominence within recent European policy frameworks, reveals a significant gap in current educational scholarship. This absence points to an urgent need for interdisciplinary research capable of developing conceptual frameworks, empirical evidence, and pedagogical models that meaningfully address the convergence of sexuality, digital media, and gender‐based harm in secondary education.

### 5.1 Implications for School Health Policy, Practice, and Equity

These findings suggest the need for more coherent educational frameworks that integrate comprehensive sexuality education, digital literacy, and gender‐based cyberviolence prevention within secondary schools. School health education should move beyond fragmented or risk‐focused approaches by supporting students in critically addressing consent, gender stereotypes, online sexual content, digital safety, and respectful relationships. From an equity perspective, policy and intervention design should be grounded in meaningful youth participation, as the active involvement of students with diverse identities and lived experiences can help operationalize an intersectional approach that starts from adolescents' own online and relational experiences and remains adaptable and scalable to diverse classroom contexts. This includes attention to differences related to gender identity, sexual orientation, ethnicity, culture, religion, socioeconomic background, disability, and other axes of inequality. Although such complexities are difficult to fully anticipate in the design of interventions, they are essential for school‐based policies and practices to be genuinely inclusive and effective.

### 5.2 Limitations

This scoping review was limited to peer‐reviewed literature, excluding gray literature and policy documents, and relied on databases that prioritize internationally indexed, predominantly English‐language publications. As a result, relevant studies published in other European languages may have been overlooked, suggesting that a multilingual approach could offer a more comprehensive understanding of the intersection between comprehensive sexuality education and digital literacy across Europe.

## 6 Conclusions

This scoping review highlights the urgent need for integrated, evidence‐based educational strategies to address gender‐based cyberviolence in European secondary schools. Although some research covers aspects of Comprehensive Sexuality Education (CSE), Digital Literacy (DL), or cyberviolence, few studies explore their intersection or translate insights into school‐based practices aligned with EU policy. The limited academic attention to gendered digital harms, combined with fragmented CSE implementation and narrow DL approaches, points to a disconnect between the complexity of these challenges and current educational responses. Addressing this gap requires interdisciplinary, intersectional research and policies that embed CSE and DL into curricula, with a focus on power, consent, and digital behaviors, crucial for ensuring safe and inclusive education for adolescents.

## Conflicts of Interest

The authors declare no conflicts of interest.

## Data Availability

The data that support the findings of this study are available from the corresponding author upon reasonable request.

## References

[1] International Planned Parenthood Federation , IPPF Framework for Comprehensive Sexuality Education (International Planned Parenthood Federation, 2010), https://youthconnect.ppfn.org/wp‐content/uploads/2021/01/ippf_framework_for_comprehensive_sexuality_education.pdf.

[2] United Nations Educational, Scientific and Cultural Organization , Joint United Nations Programme on HIV/AIDS , United Nations Children's Fund , United Nations Entity for Gender Equality and the Empowerment of Women , and World Health Organization , International Technical Guidance on Sexuality Education: An Evidence‐Informed Approach (United Nations Educational, Scientific and Cultural Organization, 2018), 10.54675/UQRM6395.

[3] E. Miedema , M. L. J. Le Mat , and F. Hague , “But Is It Comprehensive? Unpacking the “Comprehensive” in Comprehensive Sexuality Education,” Health Education Journal 79, no. 7 (2020): 747–762, 10.1177/0017896920915960.

[4] E. Ketting , L. Brockschmidt , and O. Ivanova , “Investigating the “C” in CSE: Implementation and Effectiveness of Comprehensive Sexuality Education in the WHO European Region,” Sex Education 21, no. 2 (2021): 133–147, 10.1080/14681811.2020.1766435.

[5] European Parliament and Council of the European Union , “Directive (EU) 2024/1385 of 14 May 2024 on Combating Violence Against Women and Domestic Violence,” Official Journal of the European Union (2024), https://eur‐lex.europa.eu/eli/dir/2024/1385/oj/eng.

[6] M. Dragiewicz , J. Burgess , A. Matamoros‐Fernández , et al., “Technology‐Facilitated Coercive Control: Domestic Violence and the Competing Roles of Digital Media Platforms,” Feminist Media Studies 18, no. 4 (2018): 609–625, 10.1080/14680777.2018.1447341.

[7] N. Henry and A. Powell , “Technology‐Facilitated Sexual Violence: A Literature Review of Empirical Research,” Trauma, Violence & Abuse 19, no. 2 (2018): 195–208, 10.1177/1524838016650189. 27311818

[8] M. Dragiewicz , D. Woodlock , B. Harris , and C. Reid , “Technology‐Facilitated Coercive Control,” in The Routledge International Handbook of Violence Studies, 1st ed., ed. W. S. DeKeseredy , C. M. Rennison , and A. K. Hall‐Sanchez (Routledge, 2019), 244–253, 10.4324/9781315270265-23.

[9] H. Al‐Alosi , “Cyber‐Violence: Digital Abuse in the Context of Domestic Violence,” UNSW Law Journal 40, no. 4 (2017): 1573–1603.

[10] N. Henry and A. Powell , “Technology‐Facilitated Sexual Violence: A Literature Review of Empirical Research,” Trauma, Violence & Abuse 19, no. 2 (2019): 195–208, 10.1177/1524838016650189. 27311818

[11] R. L. Abreu and M. C. Kenny , “Cyberbullying and LGBTQ Youth: A Systematic Literature Review and Recommendations for Prevention and Intervention,” Journal of Child and Adolescent Trauma 11, no. 1 (2018): 81–97, 10.1007/s40653-017-0175-7. 32318140 PMC7163911

[12] E. L. Backe , P. Lilleston , and J. McCleary‐Sills , “Networked Individuals, Gendered Violence: A Literature Review of Cyberviolence,” Violence and Gender 5, no. 3 (2018): 135–146, 10.1089/vio.2017.0056.

[13] A. Amadori , A. G. Real , A. Brighi , and S. T. Russell , “An Intersectional Perspective on Cyberbullying: Victimization Experiences Among Marginalized Youth,” Journal of Adolescence 97, no. 4 (2025): 931–940, 10.1002/jad.12466. 39827373 PMC12128902

[14] J. Hearn , M. Hall , R. Lewis , and C. Niemistö , “The Spread of Digital Intimate Partner Violence: Ethical Challenges for Business, Workplaces, Employers and Management,” Journal of Business Ethics 187, no. 4 (2023): 695–711, 10.1007/s10551-023-05463-4.

[15] B. Harris and L. Vitis , “Digital Intrusions: Technology, Spatiality and Violence Against Women,” Journal of Gender Based Violence 4, no. 3 (2020): 325–341, 10.1332/239868020X15986402363663.

[16] M. Sorochinski and E. Borukhov , “Digital Shadows: Confronting the Rise of Technology‐Facilitated Sexual Violence and the Quest for Systemic Solutions,” Contemporary Justice Review 27, no. 2–3 (2024): 116–131, 10.1080/10282580.2024.2363429.

[17] European Institute for Gender Equality , Combating Cyber Violence Against Women and Girls (European Institute for Gender Equality, 2022), https://eige.europa.eu/publications‐resources/publications/combating‐cyber‐violence‐against‐women‐and‐girls.

[18] European Union Agency for Fundamental Rights , Violence Against Women: An EU‐Wide Survey—Results at a Glance (Publications Office of the European Union, 2014), 10.2811/60683.

[19] Plan International, Girls Get Equal , Free to be Online? Girls' and Young Women's Experiences of Online Harassment (Plan International, 2020), https://plan‐international.org/publications/free‐to‐be‐online/.

[20] European Union Agency for Fundamental Rights , Challenges to Women's Human Rights in the EU: Gender Discrimination, Sexist Hate Speech and Gender‐Based Violence Against Women and Girls (Publications Office of the European Union, 2017), https://fra.europa.eu/sites/default/files/fra_uploads/fra‐2017‐challenges‐to‐women‐human‐rights_en.pdf.

[21] Y. Chen , K. Mendes , C. Gosse , J. Hodson , and G. Veletsianos , “Canadian Gender‐Based Violence Prevention Programs: Gaps and Opportunities,” Violence Against Women 31, no. 10 (2024): 2682–2704, 10.1177/10778012241259727. 38859753 PMC12171044

[22] L. Saletti‐Cuesta , L. Aizenberg , E. Torres , and L. F. Sánchez , “Elaboración de escalas de Valoración de Obstáculos y Medidas Para Abordar la Violencia de género en las Escuelas,” Interdisciplinaria: Revista de Psicología y Ciencias Afines 39, no. 1 (2021): 223–239, 10.16888/interd.2022.39.1.14.

[23] R. Parker , K. Wellings , and J. V. Lazarus , “Sexuality Education in Europe: An Overview of Current Policies,” Sex Education 9, no. 3 (2009): 227–242, 10.1080/14681810903059060.

[24] European Commission , Sexuality Education Across the European Union: An Overview (Directorate‐General for Employment, Social Affairs and Inclusion, 2020), https://ec.europa.eu/social/BlobServlet?docId=23654&langId=en.

[25] J. Cassar , “Sun, Sea, and Sex: A Comparative Study of Sexuality Education Policies in Southern Europe,” in Social Welfare Issues in Southern Europe (Taylor & Francis, 2022), 140–159, 10.4324/9780429262678-11.

[26] C. Trivelli Diaz , “Researching Youth Voices on Comprehensive Sexuality Education: A Literature Review of Qualitative Studies,” Encyclopaideia 28, no. 70 (2024): 19–33, 10.6092/ISSN.1825-8670/18974.

[27] Internet Matters , Me, Myself and AI: Understanding and Safeguarding Children's Use of AI Chatbots (Internet Matters, 2025), https://www.internetmatters.org/wp‐content/uploads/2025/07/AI‐Chatbots‐Report‐V5‐Final‐Version.pdf.

[28] M. Bonnar , I Thought He Was Going to Tear Chunks Out of My Skin (BBC News, 2020), https://www.bbc.co.uk/news/uk‐scotland‐51967295.

[29] European Commission , Digital Education Action Plan 2021–2027: Resetting Education and Training for the Digital Age (European Commission, 2020), https://education.ec.europa.eu/focus‐topics/digital‐education/action‐plan.

[30] D. Ging , “Alphas, Betas, and Incels: Theorizing the Masculinities of the Manosphere,” Men and Masculinities 22, no. 4 (2019): 638–657, 10.1177/1097184X17706401.

[31] C. Baker , D. Ging , and M. B. Andreasen , Recommending Toxicity: The Role of Algorithmic Recommender Functions on YouTube Shorts and TikTok in Promoting Male Supremacist Influencers (Dublin City University Anti‐Bullying Centre, 2024), https://antibullyingcentre.ie/wp‐content/uploads/2024/04/DCU‐Toxicity‐Full‐Report.pdf.

[32] PRISMA‐ScR , PRISMA Extension for Scoping Reviews (PRISMA‐ScR) (PRISMA Statement, 2020), https://www.prisma‐statement.org/scoping.

[33] European Commission , Union of Equality: LGBTIQ Equality Strategy 2026–2030 (European Commission, 2026), https://commission.europa.eu/strategy‐and‐policy/policies/justice‐and‐fundamental‐rights/combatting‐discrimination/lesbian‐gay‐bi‐trans‐intersex‐equality/lgbtiq‐equality‐strategy_en.

[34] S. Boer , Ö. Erdem , H. de Graaf , and H. Götz , “Prevalence and Correlates of Sext‐Sharing Among a Representative Sample of Youth in The Netherlands,” Frontiers in Psychology 12 (2021): 655796, 10.3389/fpsyg.2021.655796. 34040564 PMC8143518

[35] E.‐G. Rojas‐Estrada , A. Vizcaíno‐Verdú , and M. Bonilla del Río , “[Studies Included] Sexual (Mis)information: Pornography and Adolescence in the Digital Space,” (2024), 10.6084/m9.figshare.25331491.v2.

[36] J. M. Sill , ““I Wouldn't Have Ever Known, if It Wasn't for Porn”—LGBT+ University Students' Experiences of Sex and Relationships Education, a Retrospective Exploration,” Sex Education 23, no. 4 (2023): 379–392, 10.1080/14681811.2022.2036604.

[37] G. Testa , G. Mestre‐Bach , C. Chiclana Actis , and M. N. Potenza , “Problematic Pornography Use in Adolescents: From Prevention to Intervention,” Current Addiction Reports 10 (2023): 210–218, 10.1007/s40429-023-00469-4.

[38] L. Vandenbosch and J. M. F. van Oosten , “The Relationship Between Online Pornography and the Sexual Objectification of Women: The Attenuating Role of Porn Literacy Education,” Journal of Communication 67 (2017): 1015–1036, 10.1111/jcom.12341.

[39] K. Dawson , S. Nic Gabhainn , M. Willis , and P. MacNeela , “Development of a Measure to Assess What Young Heterosexual Adults Say They Learn About Sex From Pornography,” Archives of Sexual Behavior 51, no. 2 (2022): 1257–1269, 10.1007/s10508-021-02059-9. 34761345 PMC8888499

[40] L. Cegolon , M. Bortolotto , S. Bellizzi , A. Cegolon , G. Mastrangelo , and C. Xodo , “Birth Control Knowledge Among Freshmen of Four Italian Universities,” Scientific Reports 10, no. 1 (2020): 16466, 10.1038/s41598-020-72200-6. 33020531 PMC7536290

